# Coccidiomycosis infection of the patella mimicking a neoplasm – two case reports

**DOI:** 10.1186/1471-2342-14-8

**Published:** 2014-02-18

**Authors:** Yi-Chen Li, George Calvert, Christopher J Hanrahan, Kevin B Jones, Robert Lor Randall

**Affiliations:** 1 Department of Orthopaedic Surgery, E-DA Hospital/I-Shou University, Kaohsiung, Taiwan; 2 Division of Orthopaedic Surgery, Department of Surgery, City of Hope National Medical Center, Duarte, California, USA; 3 Department or Radiology, University of Utah School of Medicine, Salt Lake City, Utah, USA; 4 Sarcoma Services, Department of Orthopaedics, Huntsman Cancer Institute, University of Utah School of Medicine, Salt Lake City, Utah, USA

**Keywords:** Coccidioidomycosis, Patella, Infection, Lytic lesion, Computed tomography, MRI

## Abstract

**Background:**

Coccidioidomycosis is an endemic fungal infection in the southwestern of United States. Most infections are asymptomatic or manifest with mild respiratory complaints. Rare cases may cause extrapulmonary or disseminated disease. We report two cases of knee involvement that presented as isolated lytic lesions of the patella mimicking neoplasms.

**Case Presentation:**

The first case, a 27 year-old immunocompetent male had progressive left anterior knee pain for four months. The second case was a 78 year-old male had left anterior knee pain for three months. Both of them had visited general physicians without conclusive diagnosis. A low attenuation lytic lesion in the patella was demonstrated on their image studies, and the initial radiologist’s interpretation was suggestive of a primary bony neoplasm. The patients were referred for orthopaedic oncology consultation. The first case had a past episode of pulmonary coccioidomycosis 2 years prior, while the second case had no previous coccioidal infection history but lived in an endemic area, the central valley of California. Surgical biopsy was performed in both cases due to diagnostic uncertainty. Final pathologic examination revealed large thick walled spherules filled with endospores establishing the final diagnosis of extrapulmonary coccidioidomycosis.

**Conclusions:**

Though history and laboratory findings are supportive, definitive diagnosis still depends on growth in culture or endospores identified on histology. We suggest that orthopaedic surgeons and radiologists keep in mind that chronic fungal infections can mimic osseous neoplasm by imaging.

## Background

Coccidioidomycosis is an endemic fungal infection in the southwestern United States, Mexico, and some areas of Central and South America [1]. It was first described by an Argentinean medical student, Alejandro Posadas, in 1892 [2]. Infection occurs following the inhalation of *Coccidioides immitis* (endemic to California) or *posadasii* (endemic to other regions mentioned above) spores. It has an estimated incidence of over 100,000 cases annually in the U.S. with most of these infections (one-half to two-thirds) manifesting subclinically or with only mild, isolated respiratory complaints [3-6]. Rare cases (1 ~ 5%) may cause extrapulmonary or disseminated disease [1,5,7,8]. Skin, lymph node, musculoskeletal, and central nervous system have been described as sites of dissemination [1,7,9]. We present two cases of coccidiomycosis mimicking a primary neoplasm of the patella in healthy, immunocompetent males. While coccidioidomycosis is known to involve the skeleton, we are unaware of a case that has presented as a primary lesion of the patella mimicking a neoplasm.

## Case presentation

### Case #1

A healthy 27 year-old immunocompetent male was referred for orthopaedic oncology evaluation of a possible neoplasm involving the inferior pole of his left patella. The man described a four month history of progressive anterior knee pain and swelling. Suspecting a knee infection, his orthopaedic surgeon performed two separate aspirations for culture, both of which were negative for any microorganisms including fungus. Radiographs demonstrated a lucent lesion in the inferior pole of the patella, and a CT and MRI of the knee were ordered. The initial radiologist’s interpretation was suggestive of a primary bony neoplasm, and orthopaedic oncologic consultation was obtained.

The patient presented to our clinic with left anterior knee pain and limited range of motion. He reported an episode of “Valley Fever” two years previously when he worked in the Central Valley of California. The isolated pulmonary infection was ultimately confirmed to be *Coccidioides spp.* by sputum culture. He was treated with a 6-month course of oral fluconazole with complete resolution of all symptoms. Notably, he never had any musculoskeletal complaints during that episode. On review of systems he described a 10 to 15 pound weight loss over the past month with intermittent nocturnal fevers and sweats.

On physical examination, the patient walked with an antalgic gait. There was a left knee effusion and warmth. He had localized tenderness along the inferior pole of his patella. His passive range of motion was limited from 5 to 95 degree. There was no palpable mass about the affected knee.

**Figure 1:**
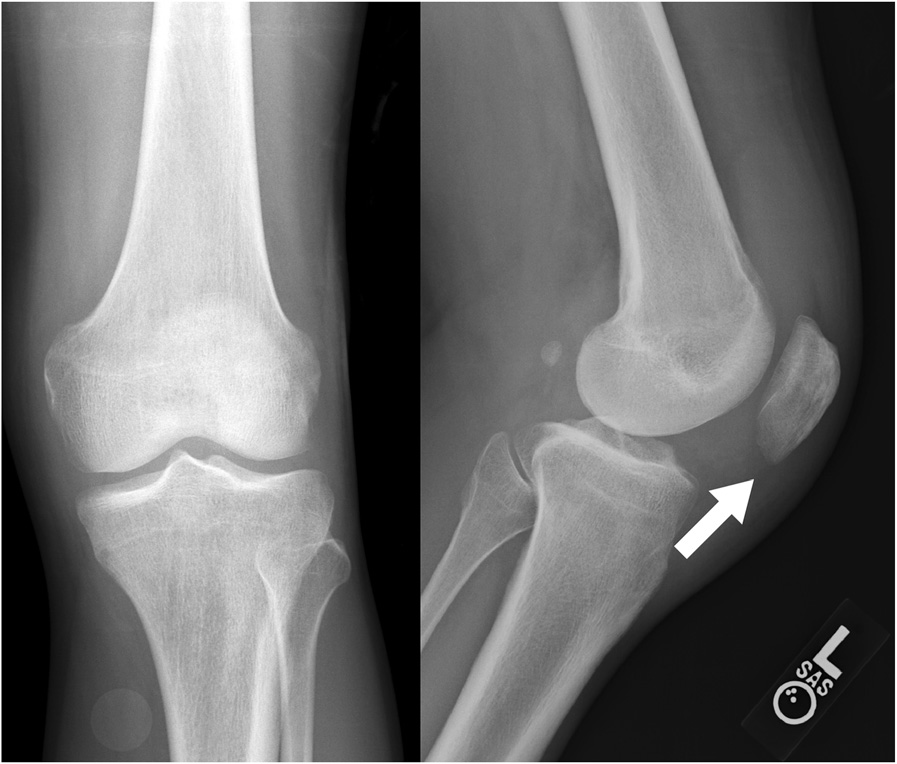
**AP and lateral radiographs of the left knee demonstrate lucency involving the inferomedial patella with questionable focal lesion (white arrow) and a joint effusion.** No soft tissue calcification is present.

**Figure 2:**
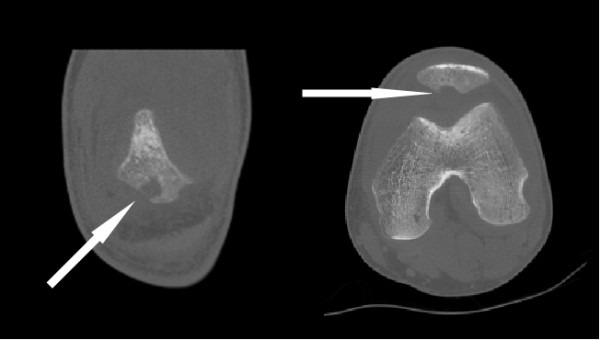
Coronal (left) and axial (right) images from a noncontrasted CT scan in bone algorithm (Width 2500, center 350; coronal 0.63 mm reconstruction; axial 1.25 mm slice) demonstrate more clearly, a focal area of osteolysis involving the inferior pole of the patella (white arrows).

**Figure 3:**
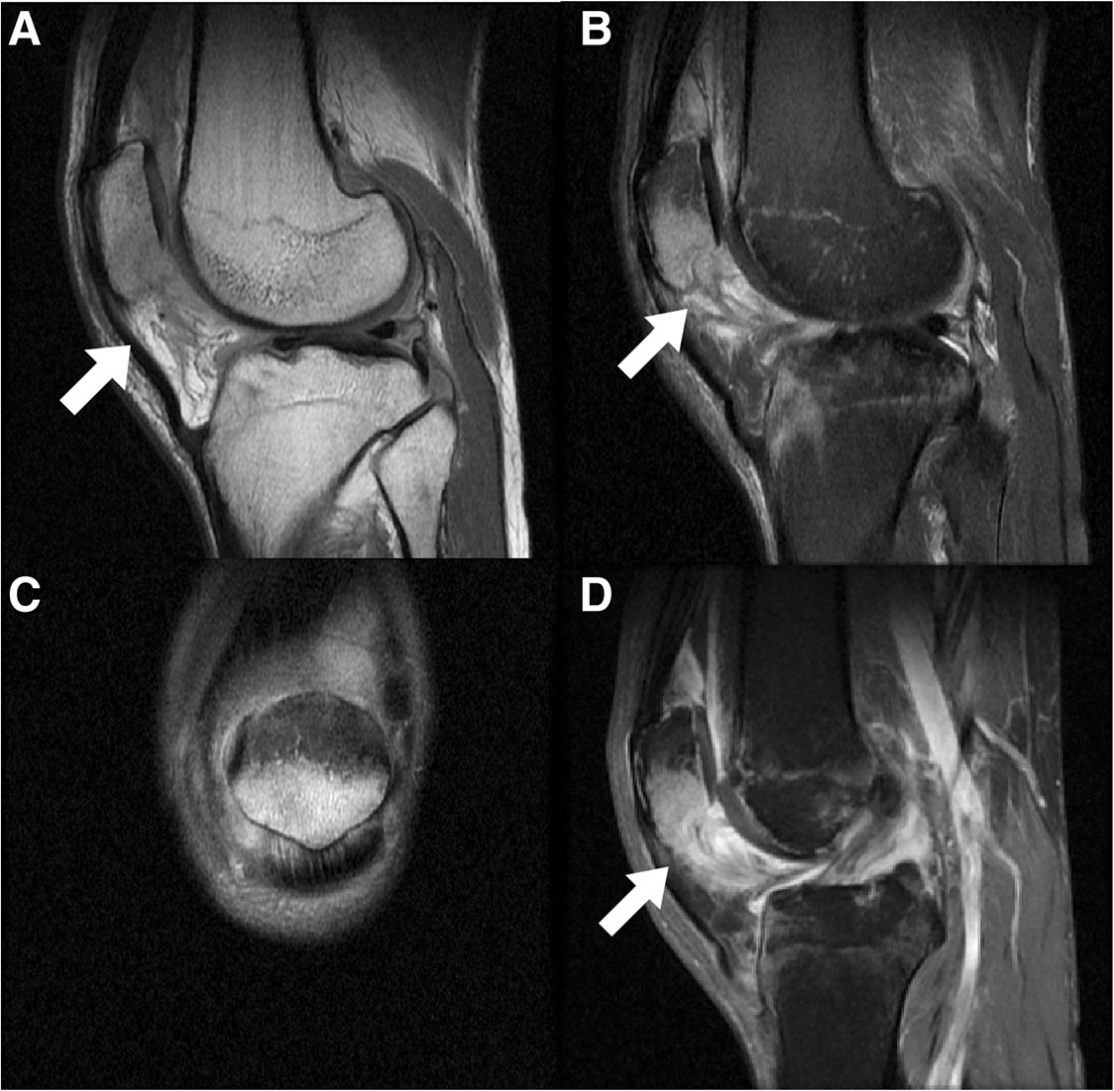
**A contrasted knee MRI had been performed at an outside facility (A: Sagittal T1 proton density (TE 34; TR 1967); B: Sagittal T2 with fat saturation (TE 54; TR 3067); C: Coronal T2 with fat saturation (TE 59; TR 2733); D: Gadolinium enhanced Sagittal T1 with fat saturation (TE 10; TR 533)).** This demonstrates knee joint effusion with extensive synovitis involving Hoffa’s fat (arrow: A, B, D) and continuity with the osteolytic area within the inferior pole of the patella. There is abundant adjacent bone marrow edema within the patella (B, C) and proximal tibia (B).

Radiographs of his left knee demonstrated a joint effusion and lucency involving the inferior pole of patella, which was visualized best on the lateral view (Figure 1). On CT performed at an outside hospital, a low attenuation lytic lesion was more clearly defined without associated soft tissue calcification (Figure 2). The outside MRI demonstrated knee joint effusion with extensive synovitis extending into Hoffa’s fat and continuity with the low attenuation area within the inferior pole of the patella along with extensive marrow edema (Figure 3) of the proximal tibia and patella.

Laboratory data at our institute revealed a mild elevation in the C-reactive protein level of 3.0 (normal range: 0 ~ 0.8) and the Westergren erythrocyte sedimentation rate of 22 (normal value: 0 ~ 10). He had a normal white blood cell count (6.93 × 10^3^) with a normal polymorphonuclear cell differential (66%) and a mildly elevated monocytic differential (9%, normal monocyte differential: 2% ~ 8%). His coccidioides antibody serology tests were positive by complement fixation (CF) method (1:1024, normal value: <1:2) as well as by the enzyme immunoassay method to IgG (IgG antibody: 9.6, normal value: ≤0.9) and IgM (IgM antibody: 1.2, normal value ≤ 0.9) antibodies.

Knee infection related to the previous coccidiomycosis exposure was highly suspected despite the two prior negative cultures on aspiration. He therefore underwent arthroscopic examination and biopsy of the left knee which revealed inflamed synovium throughout the knee with soft necrotic bone at the inferior pole of the patella. Frozen section analysis of the patellar bone revealed abundant chronic inflammatory cells without evidence of malignancy. Extensive open debridement over the inferior patella was performed. Postoperatively, his pain was immediately better, and his fevers and night sweats resolved.

**Figure 4:**
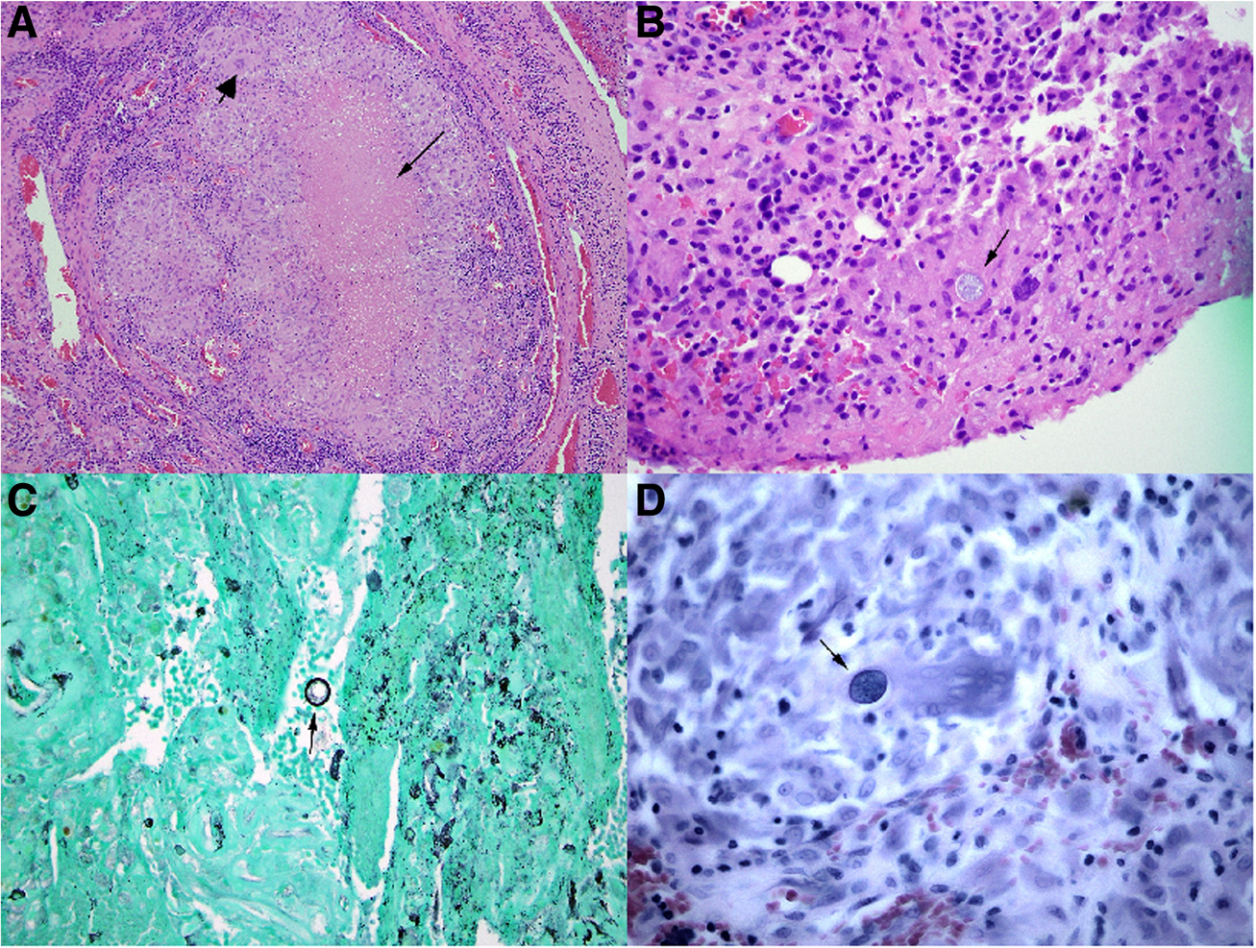
**Histology with Hematoxilin and Eosin (H&E) staining.** A low power objective (**A**, 10X) showed areas of synovium with necrosis (thin arrow) and granulomatous inflammation (thick arrowhead). At a higher magnification (**B**, H&E stain, 40X), a spherule (arrow) containing endospores could be clearly identified. Spherules and endospores are also visible by the Gomori’s methenamine silver (GMS) stain (**C**, 40X) and by the acid fast bacillus (AFB) stain (**D**, 60X). The size and morphology of these structures is consistent with *Coccidioides immitis*.

Final pathologic examination revealed large thick walled spherules filled with endospores establishing the final diagnosis of extrapulmonary coccidioidomycosis (Figure 4). The patient was treated with an oral antifungal regimen (oral fluconazole 800 mg as loading dose at first day, followed by 400 mg daily for at least one year) per infectious disease specialist recommendations. At six week follow-up, he had complete resolution of symptoms and normal knee range of motion.

### Case #2

An active 78 year old immunocompetent male was referred for orthopaedic oncology evaluation of a left patellar bone lesion. The patient had experienced three months of increasing left anterior knee pain without any history of antecedent trauma. He had no history of fevers, chills, malaise, or weight loss. His only medical issues were cardiac arrhythmia requiring pacemaker placement and benign prostatic hypertrophy. The referring physician had attempted conservative treatment with a corticosteroid injection without improvement of the knee pain. The patient lives in the central valley of California, but had no known history of coccidioidomycosis.

Physical examination was notable for exquisite tenderness of the superolateral border of the patella. Gait, alignment, and knee range of motion were normal. No inguinal or popliteal lymph nodes were palpable. There was no knee effusion.

**Figure 5:**
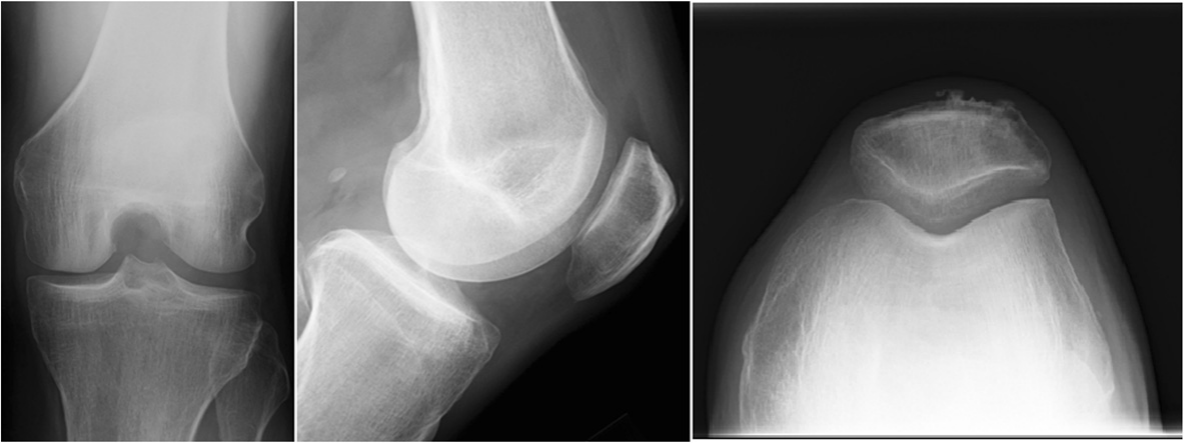
AP, lateral, and sunrise radiographs of the knee demonstrate a lucent lesion of the superolateral patella.

**Figure 6:**
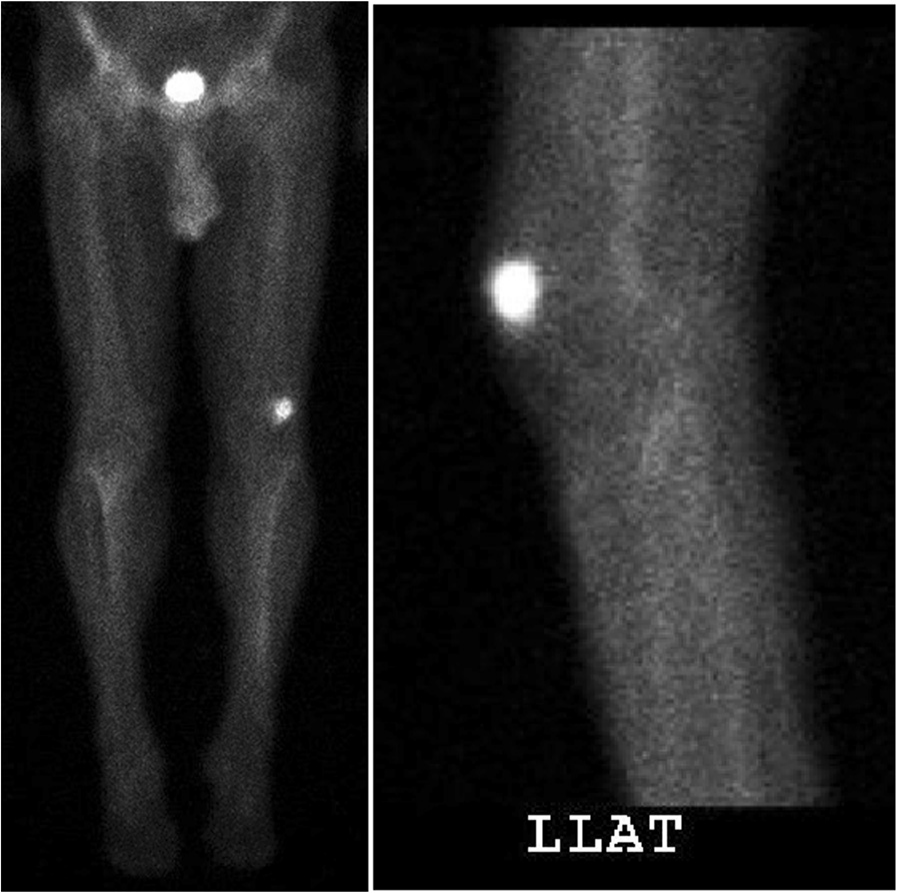
**AP and coned down lateral images from a technetium-99 m bone scan reveals increased uptake within the isolated left superolateral patella lesion.** It also appears to have a narrow zone of transition on the lateral view.

**Figure 7:**
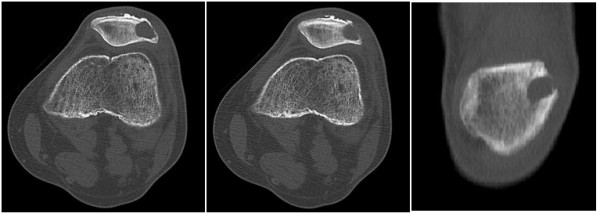
**Computed tomography in bone algorithm demonstrates the well defined, low attenuation lesion of the left patella.** A breach of the lateral patellar cortex is noted on both the axial and coronal views. No associated soft tissue mass or matrix is identified.

Radiographs revealed a lucent lesion of the superolateral patella (Figure 5). Bone scan demonstrated increased uptake of the lesion without any other areas of abnormal skeletal uptake (Figure 6). Computed tomography further demonstrated the low attenuation lesion without associated soft tissue mass or knee effusion (Figure 7). Notably, the imaging reports stated that there was a high suspicion for primary bone malignancy or metastatic disease.

**Figure 8:**
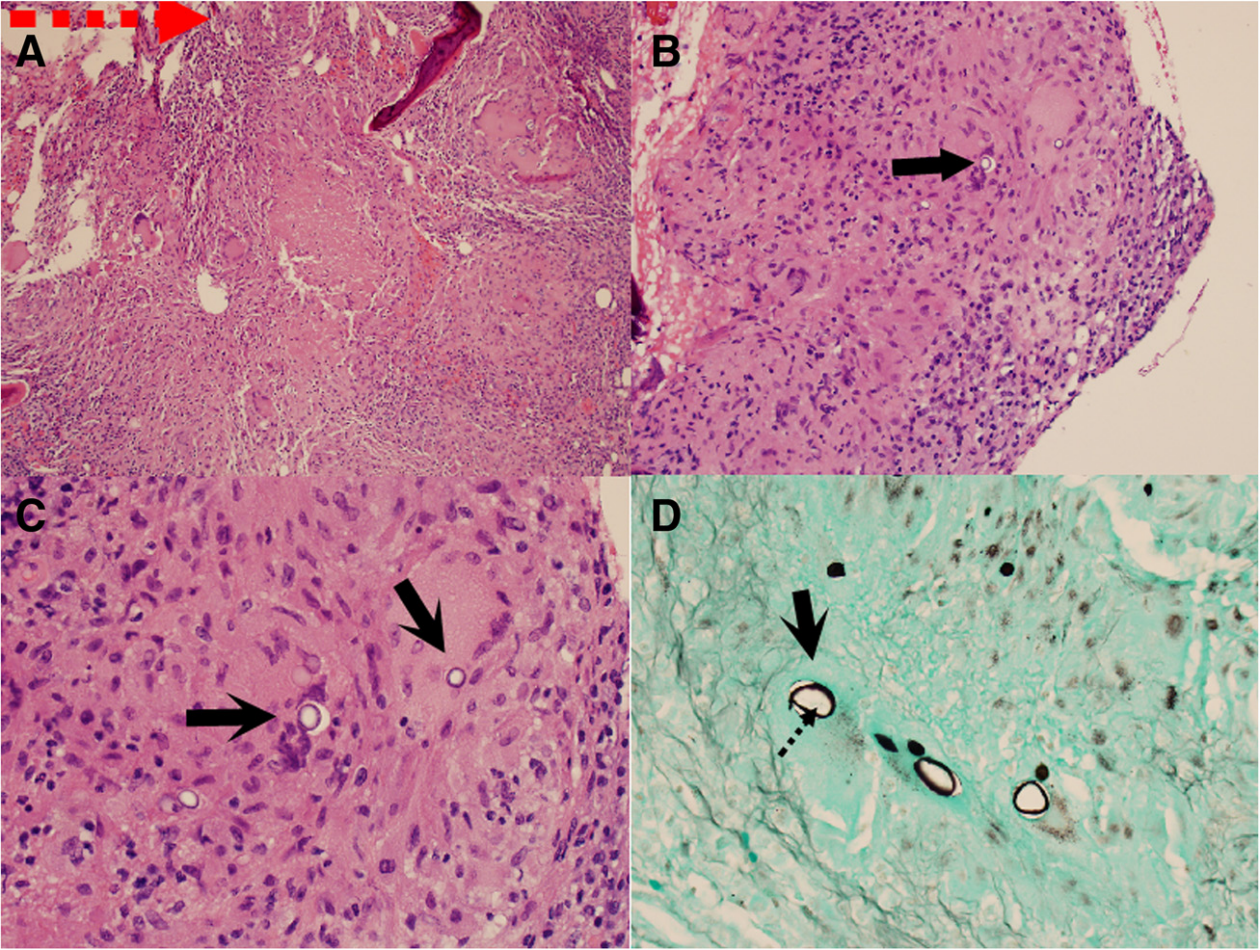
**Histology with Hematoxilin and Eosin (H&E) staining.** A low power objective (**A**, 10X) showed areas of granulomatous inflammation. At a higher magnification (**B**, H&E stain, 40X) (**C**, H&E stain, 60X), several spherules (arrows) could be clearly identified. Spherule (arrow) and endospores (dot arrow) are also visible by the Gomori’s methenamine silver (GMS) stain (**D**, 40X). The size and morphology of these structures is consistent with *Coccidioides immitis*.

Open biopsy was performed, and gross examination of tissue from the lesion was most consistent with infection. Frozen section analysis was non-diagnostic. Limited curettage of the lesion through the open biopsy tract was therefore performed. Final pathology revealed spherules filled with endospores, classic for coccidioidomycosis (Figure 8). Culture of the tissue confirmed *Coccidioides spp.* infection. Chest radiographs showed mild right hilar fullness. His coccidioides antibody serology tests were positive by complement fixation (CF) method (1:32, normal value: <1:2) as well as qualitatively by the enzyme immunoassay method to IgG but not to IgM.

The patient noted immediate improvement of his knee symptoms post-operatively. One month follow-up revealed resolution of the patellar tenderness and normal knee function. The patient was seen by an infectious disease specialist and initiated on a 6 month course of oral fluconazole.

## Conclusions

Individuals at risk for Coccidiomycosis dissemination include persons of Filipino or African ancestry, pregnant or postpartum women, and immunocompromised patients [3-5,10,11]. Musculoskeletal infection by fungal infections, including *Coccidioides spp.,* was once rare [1]. The incidence is increasing, not only in immunocompromised patients but also among healthy persons [1]. In some series it even accounts for 20% to 50% of disseminated cases [7]. The knee and the spine are the most commonly affected musculoskeletal sites [7-9,12]. Often, fungal arthritis follows a chronic indolent course of several months that may lead to delays in diagnosis and inappropriate treatments [1]. Skeletal manifestations associated with coccidioidomycosis include a chronic granulomatous process in bones, joints, and periarticular structures [1,13]. Coccidioidal arthritis should always be suspected when a patient has progressive arthritis and a history of travel to an endemic zone. Recent literature suggests that the majority of septic arthritis cases are bone coccidioidomycosis with secondary involvement of the joint [14]. A history of pulmonary disease or an abnormal chest radiograph helps confirm the diagnosis [1].

To formally establish a diagnosis of coccydiomycosis, Maves et al. proposed serologic testing, histopathologic confirmation, or growth of *Coccidioides* in culture [5]. A positive mycological culture may grow relatively rapidly after inoculation into media (e.g. 48 hours), although 5 days or longer is required to be negative [3,15]. Serologic testing plays an important role in the non-invasive diagnosis of patients with coccidioidomycosis [3,5,9,10]. The IgM antibodies appear in the acute stage, and can detect in up to 75% of primary cases. With chronic infection, IgG antibodies are found and persist in relation to the extent of disease. The sensitivity of the IgG antibody test by Szeyko et al. in 36 chronic vertebral involvement cases, yielded a sensitivity of 88%. However, sensitivity of the test was lower in immunocompromised group than for immunocompetent patients (67% vs 93%) [9]. Inflammatory markers as ESR and CRP may also suggest the diagnosis of infection, but no clear relationship to the ratios has been noted [9].

Imaging findings in musculoskeletal coccidioidocomycosis are nonspecific [8]. The most frequent radiographic findings are that of multiple lytic, punched-out lesions. Lesions with permeative or moth-eaten borders, or sclerotic margins may be identified depending on chronicity and aggressivity of infection [12]. MRI and bone scan are nonspecific [8,13]. The most common finding by MRI is hypertrophied synovium [8]. Other findings may include bone marrow edema or subchondral bone loss [8]. Distinguishing coccidioidal from tuberculous synovitis by imaging is not possible [11]. It is important to firmly establish the diagnosis before initiating what may be inappropriate treatment thereby delaying appropriate management. Despite many non-invasive techniques, synovial biopsy and culture still remains the most effective way to determine the true diagnosis [11].

Though our first patient presented with a prior history of coccidioidomycosis, failure of the culture result and previous occult lab findings were obfuscating. Our second patient had no antecedent history of fungal infection; however, he did live in an endemic area. In both cases, the presence of an isolated osteolytic lesion made the distinction between infection and tumor more challenging.

We are aware of two case reports describing chronic coccidiodomycosis of the knee [15,16]. In these cases the disease involved the synovium and cancellous bones of distal femur and proximal tibia, which were treated with joint debridement, synovectomy, and antifungal drugs. To our knowledge, these are the first reported cases of patellar involvement in an immunocompetent adult. We suggest that orthopaedic surgeons and radiologists keep in mind that the chronic fungal infections such as this can mimic a bone or soft tissue neoplasm.

### Consent

“Written informed consent was obtained from the patients for publication of these case reports and any accompanying images. A copy of the written consents is available for review by the Editor-in-Chief of this journal”.

## Competing interests

Financial competing interests

In the past five years have you received reimbursements, fees, funding, or salary from an organization that may in any way gain or lose financially from the publication of this manuscript, either now or in the future: **No**

Is such an organization financing this manuscript (including the article-processing charge): **No**

Do you hold any stocks or shares in an organization that may in any way gain or lose financially from the publication of this manuscript, either now or in the future: **No**

Do you hold or are you currently applying for any patents relating to the content of the manuscript: **No**

Have you received reimbursements, fees, funding, or salary from an organization that holds or has applied for patents relating to the content of the manuscript: **No**

Do you have any other financial competing interests: **No**

Non-financial competing interests

Are there any non-financial competing interests (political, personal, religious, ideological, academic, intellectual, commercial or any other) to declare in relation to this manuscript: **No**

All authors reported that they have no financial interests or relationships that pose a potential conflict of interest with this article.

## Authors’ contributions

YCL, GC, and RLR contributed to all aspects of the manuscript. CJH and KBJ participated in data analysis, drafting and revision of the manuscript, and final approval of the manuscript before publication. All authors read and approved the final manuscript.

## Pre-publication history

The pre-publication history for this paper can be accessed here:

http://www.biomedcentral.com/1471-2342/14/8/prepub
